# Family and informal caregivers’ acceptance, concerns and expectations regarding artificial intelligence-enabled dementia care: a protocol for qualitative evidence synthesis

**DOI:** 10.1371/journal.pone.0360163

**Published:** 2026-10-07

**Authors:** Fang Wang, Jianqiong Li, Yingjie Shen

**Affiliations:** 1 School of Nursing, Chongqing Three Gorges Medical College, Chongqing, China; 2 Department of Nursing Science, Faculty of Medicine, University of Malaya, Kuala Lumpur, Malaysia; KPC Medical College and Hospital, INDIA

## Abstract

**Introduction:**

Artificial intelligence (AI) is increasingly being integrated into dementia care to support assessment, monitoring, decision-making and personalized care. However, the successful implementation of AI-enabled dementia care depends not only on technological capabilities but also on the acceptance, trust and engagement of family and informal caregivers. Existing qualitative evidence remains fragmented and has not comprehensively synthesized caregivers’ perspectives regarding AI-enabled dementia care. This qualitative evidence synthesis aims to address this gap by exploring caregivers’ acceptance, concerns and expectations.

**Methods and analysis:**

This qualitative evidence synthesis will be conducted in accordance with the Joanna Briggs Institute (JBI) methodology for qualitative systematic reviews and reported following the Preferred Reporting Items for Systematic Review and Meta-Analysis Protocols (PRISMA-P) statement. Studies will be identified through systematic searches of PubMed, Embase, CINAHL, PsycINFO and Web of Science from database inception to December 2026. Qualitative and mixed-methods studies exploring family and informal caregivers’ perspectives, experiences, acceptance, concerns or expectations regarding AI-enabled dementia care will be included. Study selection and methodological quality appraisal using the JBI Critical Appraisal Checklist for Qualitative Research will be conducted independently by two reviewers. Data will be extracted using a predefined form and synthesized using Thomas and Harden’s thematic synthesis approach. Confidence in the review findings will be assessed using the Grading of Recommendations Assessment, Development and Evaluation-Confidence in the Evidence from Reviews of Qualitative Research (GRADE-CERQual) approach.

**Ethics and dissemination:**

Ethical approval is not required. Findings will be disseminated through peer-reviewed publication.

**Trial registration:**

PROSPERO registration number: CRD420261470957.

## Introduction

Dementia is a progressive neurodegenerative condition that affects more than 58 million people worldwide and is a leading cause of disability, dependency and long-term care needs among older adults [1]. As populations age, the growing prevalence of dementia is placing substantial pressure on healthcare systems, social care services and families, highlighting the urgent need for innovative approaches to support high-quality, person-centered dementia care [2]. In recent years, artificial intelligence (AI) has emerged as a promising technology with the potential to transform dementia care across the disease trajectory [3]. AI-enabled technologies, including machine learning algorithms, clinical decision support systems, conversational AI, intelligent monitoring systems and socially assistive robots, are increasingly being integrated into dementia care to support early detection, personalized care planning, symptom monitoring, risk prediction and daily care management [4–6]. These technologies have the potential to enhance care efficiency, facilitate timely decision-making, promote independent living and improve care coordination across home, community and healthcare settings [7,8]. As AI applications continue to expand beyond diagnosis into the delivery and management of dementia care, understanding the factors that influence their successful implementation in real-world care settings has become an increasingly important priority for researchers, clinicians, technology developers and policymakers [9].

Family and informal caregivers are central to the delivery of dementia care due to the inaccessibility of costly professional care, lack of insurance support, and cultural barriers [10]. As dementia progresses and cognitive impairment worsens, caregivers frequently assume increasing responsibility for monitoring health status, managing behavioral and psychological symptoms, communicating with healthcare professionals and making care-related decisions on behalf of the person living with dementia [11]. Consequently, they are among the primary end-users and decision-makers regarding the adoption and sustained use of AI-enabled technologies in dementia care. Although AI has the potential to enhance caregiving by improving care coordination, reducing caregiving burden and supporting timely decision-making, its successful implementation depends not only on technological capability but also on caregivers’ willingness to accept, trust and integrate these technologies into routine care [12]. Family and informal caregivers may simultaneously perceive AI as a source of practical support while expressing concerns regarding privacy, data security, ethical implications, reduced human interaction, accountability and the potential replacement of person-centered care [13,14]. They may also hold diverse expectations regarding how AI should complement, rather than replace, human caregiving [15]. Therefore, understanding caregivers’ acceptance, concerns and expectations is essential for developing, implementing and governing AI-enabled dementia care that is clinically meaningful, ethically responsible and responsive to the needs and preferences of families.

A growing body of qualitative research has explored caregivers’ experiences with digital technologies designed to support dementia care [16]. Existing evidence syntheses have examined specific interventions, such as socially assistive robots and supportive mobile applications, providing valuable insights into caregivers’ experiences, perceived benefits and implementation challenges associated with individual technologies [15,17]. However, these reviews have largely focused on single technological modalities rather than the broader and rapidly evolving landscape of artificial intelligence-enabled dementia care [18,19]. AI-enabled technologies differ fundamentally from conventional digital interventions because they increasingly incorporate autonomous decision-making, predictive analytic, adaptive learning and data-driven personalization, giving rise to distinctive practical, ethical and governance considerations [5,20,21]. Consequently, findings derived from reviews of individual technologies cannot fully explain how family and informal caregivers understand, evaluate and respond to AI-enabled dementia care across different applications and care settings. Moreover, while an increasing number of qualitative studies have explored caregivers’ views on AI-supported dementia care, the evidence remains fragmented across diverse technologies, contexts and study populations [12,17]. To date, few qualitative evidence has comprehensively integrated family and informal caregivers’ acceptance, concerns and expectations regarding AI-enabled dementia care. As a result, there remains limited understanding of the factors influencing caregivers’ acceptance of AI, the concerns that may hinder its implementation, and the expectations that should inform the development of safe, trustworthy and caregiver-centered AI solutions for dementia care.

A qualitative evidence synthesis is well suited to addressing this evidence gap because it enables the systematic integration and interpretation of caregivers’ lived experiences, perspectives and contextual understandings across diverse qualitative studies [22]. By synthesizing qualitative evidence, this review will move beyond describing individual study findings to generate a more comprehensive understanding of the factors shaping family and informal caregivers’ acceptance, concerns and expectations regarding AI-enabled dementia care. Such evidence is essential for informing the development of caregiver-centered AI technologies that are not only technically effective but also acceptable, trustworthy and ethically responsible in real-world care settings. The findings of this review may also support healthcare professionals, technology developers and policymakers in designing, implementing and governing AI-enabled dementia care that better aligns with the needs, values and preferences of families caring for people living with dementia. In addition, the review will identify gaps in the current qualitative evidence base and provide directions for future research on the responsible integration of AI into dementia care. Therefore, this qualitative evidence synthesis aims to systematically synthesize qualitative evidence on family and informal caregivers’ acceptance, concerns and expectations regarding artificial intelligence-enabled dementia care, thereby informing the future development, implementation and governance of caregiver-centered AI in dementia care.

## Methods

### Study design and protocol

This protocol describes a qualitative evidence synthesis that aims to systematically synthesize qualitative evidence on family and informal caregivers’ acceptance, concerns and expectations regarding artificial intelligence (AI)-enabled dementia care. A qualitative evidence synthesis was selected because it enables the integration and interpretation of findings from qualitative studies to generate a comprehensive understanding of participants’ perspectives, meanings and contextual experiences across diverse settings [23]. The review will be conducted in accordance with the Joanna Briggs Institute (JBI) methodology for qualitative systematic reviews, which provides a rigorous and transparent approach for identifying, appraising, synthesizing and reporting qualitative evidence [24].

The protocol has been developed and reported in accordance with the Preferred Reporting Items for Systematic Review and Meta-Analysis Protocols (PRISMA-P) 2015 Statement [25]. The completed review will be reported in accordance with the Enhancing Transparency in Reporting the Synthesis of Qualitative Research (ENTREQ) statement to ensure comprehensive and transparent reporting of the review process and findings [26].

This review protocol has been registered with the International Prospective Register of Systematic Reviews (PROSPERO) (registration number: CRD420261470957). Any important amendments to the protocol made during the review process will be documented, justified and reported in the final publication to ensure transparency and reproducibility.

### Study status and timeline

This manuscript presents a protocol for a qualitative evidence synthesis. At the time of submission, the review has not yet commenced. The systematic literature search is planned to be conducted in October 2026. Record screening is expected to be completed by January 2027, and data extraction is expected to be completed by March 2027. Data synthesis and interpretation are expected to be completed by May 2027, with the final review results anticipated by July 2027. These dates represent the anticipated timeline and may be adjusted depending on the volume of retrieved records, the number of eligible studies, and the progress of the review.

### Eligibility criteria

Eligibility criteria were developed using the SPIDER (Sample, Phenomenon of Interest, Design, Evaluation and Research type) framework, which is considered appropriate for identifying qualitative evidence addressing participants’ experiences, perspectives and meanings [27,28].

#### Sample (S).

This review will include studies involving family and informal caregivers of people living with dementia, regardless of caregivers’ age, sex, relationship to the care recipient or care setting. Family and informal caregivers are defined as unpaid individuals, such as spouses, partners, adult children, relatives, friends or neighbors, who provide ongoing physical, emotional or practical support to a person living with dementia. Studies focusing exclusively on healthcare professionals, paid caregivers, formal care staff or people living with dementia without reporting caregivers’ perspectives will be excluded.

#### Phenomenon of Interest (PI).

The phenomenon of interest is family and informal caregivers’ acceptance, concerns and expectations regarding the use of artificial intelligence (AI)-enabled technologies in dementia care. AI-enabled technologies may include, but are not limited to, machine learning-based decision support systems, conversational AI, generative AI applications, intelligent monitoring systems, predictive analytic, socially assistive robots incorporating AI, and other AI-supported technologies used to facilitate dementia care. Studies investigating digital health technologies without AI components, conventional telehealth services or non-AI electronic health interventions will be excluded. Where studies evaluate technologies incorporating both AI and non-AI functions, they will be included only if AI-related findings can be clearly identified or constitute a substantial component of the intervention.

#### Design (D).

Primary qualitative studies using recognized qualitative data collection methods, including individual interviews, focus groups, observations or qualitative open-ended responses, will be eligible. Mixed-methods studies will also be included if qualitative findings can be extracted separately from quantitative results.

#### Evaluation (E).

Eligible studies must report qualitative findings relating to caregivers’ acceptance, perceptions, attitudes, beliefs, trust, concerns, expectations, preferences, perceived benefits, perceived challenges or other experiences associated with AI-enabled dementia care.

#### Research type (R).

This review will include peer-reviewed qualitative and mixed-methods studies published in English. Conference abstracts, editorials, commentaries, study protocols, opinion papers, dissertations, reviews and studies without accessible full texts will be excluded.

### Search strategy

A comprehensive and systematic search strategy will be developed in consultation with an experienced health sciences librarian to maximize the identification of relevant qualitative studies. The search strategy will follow the three-step approach recommended by the Joanna Briggs Institute (JBI) for qualitative systematic reviews [29]. First, an initial limited search of selected databases will be undertaken to identify relevant articles and analyze text words contained in titles and abstracts, together with index terms used to describe the articles. Second, a comprehensive search strategy will be developed and adapted for each database using the identified keywords and controlled vocabulary where appropriate. Finally, the reference lists of all included studies and relevant reviews will be screened to identify additional eligible studies.

A comprehensive systematic literature search will be conducted between 1 October 2026 and 31 December 2026. The following electronic databases will be searched from inception to January 2027: PubMed, Embase, CINAHL, PsycINFO and Web of Science. These databases were selected to ensure comprehensive coverage of the medical, nursing, psychological and interdisciplinary literature relevant to dementia care, caregiving and artificial intelligence [30]. Only studies published in English will be included because of resource constraints for translation.

The search strategy will combine controlled vocabulary (e.g., Medical Subject Headings [Mesh], where applicable) and free-text terms related to three key concepts: (1) dementia, (2) family and informal caregivers, and (3) artificial intelligence-enabled dementia care. Search terms relating to artificial intelligence will include technologies such as conversational AI, generative AI, machine learning, predictive analytic, intelligent monitoring systems and socially assistive robots incorporating AI, together with relevant synonyms and database-specific indexing terms [31]. Search terms for each concept will be combined using Boolean operators (AND/OR) [32]. Database-specific search strategies will be adapted according to the indexing system and search interface of each database. The complete search strategy for PubMed is provided in S1 Appendix and will be adapted for each database as appropriate.

To minimize publication bias and maximize the completeness of the evidence base, backward citation searching of all included studies and relevant review articles will be undertaken [33]. Where necessary, forward citation searching will also be performed using Web of Science to identify additional eligible studies [34]. If important information is unclear or incomplete, corresponding authors may be contacted for clarification. Grey literature will not be systematically searched because the review aims to synthesize peer-reviewed qualitative evidence that has undergone formal academic quality assessment.

Search results from all databases will be imported into EndNote, where duplicate records will be identified and removed prior to screening.

### Study selection

Following completion of the literature search, all retrieved records will be imported into EndNote, where duplicate records will be identified and removed. The remaining records will then be transferred to a systematic review management platform for screening.

Study selection will be conducted in two sequential stages by two reviewers working independently [35]. In the first stage, titles and abstracts will be screened against the predefined eligibility criteria developed using the SPIDER framework. Studies that clearly do not meet the inclusion criteria will be excluded. In the second stage, the full texts of all potentially eligible studies will be retrieved and independently assessed for inclusion by the same two reviewers. Reasons for excluding studies at the full-text screening stage will be documented to ensure transparency and reproducibility. Any disagreements arising during either stage of the screening process will be resolved through discussion between the two reviewers. If consensus cannot be reached, a third reviewer will be consulted to make the final decision. Prior to formal screening, a pilot screening exercise will be undertaken using a sample of retrieved records to ensure consistent interpretation and application of the eligibility criteria by all reviewers. Where necessary, the eligibility criteria will be refined to improve clarity and consistency before full screening commences. Inter-reviewer agreement during the pilot screening stage will be assessed, and any inconsistencies will be discussed to achieve a shared understanding of the eligibility criteria before formal screening begins.

The study selection process will be documented and reported using a Preferred Reporting Items for Systematic Reviews and Meta-Analyses (PRISMA) 2020 flow diagram, which will present the numbers of records identified, screened, excluded and included at each stage of the review process [36]. Fig 1 illustrates the study selection process.

**Fig 1:**
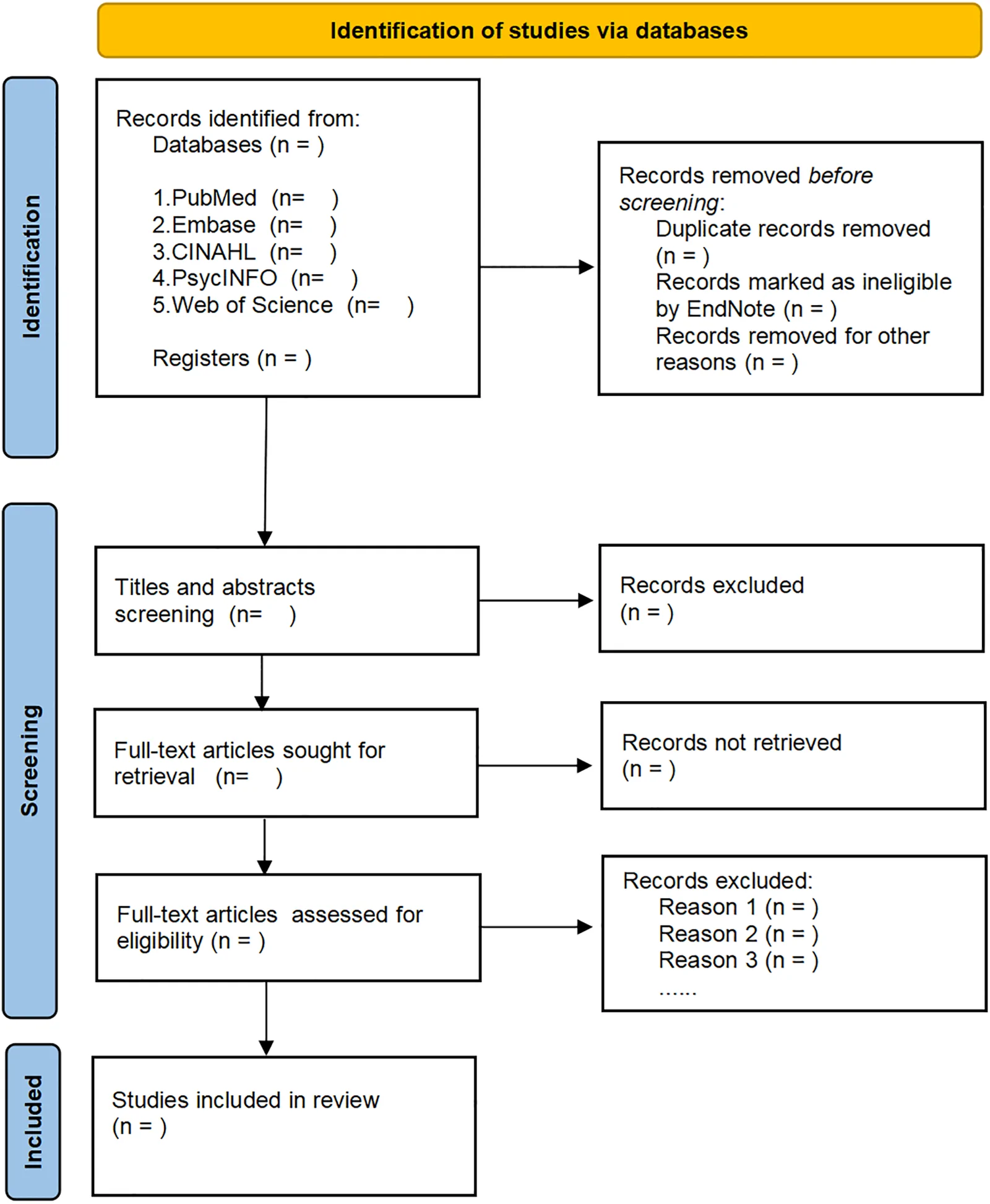
Flowchart of the literature selection process.

### Methodological quality appraisal

The methodological quality of all included studies will be independently appraised by two reviewers using the Joanna Briggs Institute (JBI) Critical Appraisal Checklist for Qualitative Research [37]. This checklist comprises ten items that assess the methodological rigour, credibility and transparency of qualitative studies, including the congruity between the research methodology, research questions, data collection methods, data analysis, interpretation of findings, researcher reflexivity, ethical considerations and the representation of participants’ voices.

Prior to formal appraisal, the reviewers will pilot the checklist on a small sample of included studies to ensure a consistent understanding and application of the appraisal criteria [38]. The reviewers will discuss any uncertainties encountered during pilot appraisal to promote consistency before the formal quality assessment. Any discrepancies in quality assessments will be resolved through discussion, and where consensus cannot be reached, a third reviewer will be consulted.

Methodological quality appraisal will be undertaken to facilitate interpretation of the review findings rather than to determine study eligibility [39]. Therefore, studies meeting the predefined eligibility criteria will not be excluded solely on the basis of methodological limitations. Instead, the results of the critical appraisal will be considered during data synthesis and in assessing the overall confidence in the review findings. The methodological quality of included studies will be summarized in S1 Table and described narratively in the final review.

### Data extraction

Data extraction will be undertaken independently by two reviewers using a standardized data extraction form developed specifically for this review (S2 Table). Prior to formal data extraction, the form will be piloted on a small number of included studies to ensure that all relevant information can be extracted consistently and comprehensively [40]. Any necessary refinements to the extraction form will be made before the full data extraction process begins.

The following information will be extracted from each included study: first author, year of publication, country, study aim, study setting, participant characteristics (including caregiver type, sample size and demographic information where available), characteristics of people living with dementia (where reported), type of AI-enabled technology, study design, qualitative methodology, data collection methods, data analysis approach, and key qualitative findings relating to caregivers’ acceptance, attitudes, beliefs, trust, concerns, expectations, perceived benefits, perceived challenges and experiences of AI-enabled dementia care. Illustrative participant quotations and authors’ interpretations supporting the reported findings will also be extracted, where available, to facilitate an in-depth understanding of the qualitative evidence. Data extraction will focus on findings presented in the Results or Findings sections of the included studies, together with the accompanying participant quotations and authors’ interpretations relevant to the review question.

Any disagreements during data extraction will be resolved through discussion between the two reviewers. Where consensus cannot be reached, a third reviewer will be consulted. If important study information is missing or unclear, attempts will be made to contact the corresponding authors for clarification. Any modifications made to the data extraction form during the review process will be documented and reported to ensure transparency and reproducibility.

### Data synthesis

Qualitative data will be synthesized using the thematic synthesis approach described by Thomas and Harden, which is widely used for integrating findings from qualitative studies in health research [41]. This approach was selected because it enables the systematic identification, comparison and interpretation of concepts across studies while preserving the contextual richness of the original qualitative findings [22]. It is particularly appropriate for exploring family and informal caregivers’ acceptance, concerns and expectations regarding AI-enabled dementia care across diverse technologies and care settings.

The synthesis will be conducted in three sequential stages [41]. First, the findings of each included study, together with the accompanying participant quotations where available, will be imported into qualitative data management software (e.g. NVivo) and coded line by line. Coding will remain inductive throughout this stage to ensure that concepts are derived from the original data rather than imposed by pre-existing theoretical frameworks. Second, codes with similar meanings will be compared and grouped into descriptive themes that summarize recurring patterns across the included studies. Third, descriptive themes will be further interpreted and refined to generate higher-order analytical themes that provide new conceptual insights into family and informal caregivers’ acceptance, concerns and expectations regarding AI-enabled dementia care.

The synthesis will be undertaken independently by two reviewers, with regular discussions throughout the analytical process to compare coding decisions, resolve discrepancies and refine the developing thematic framework. Any disagreements that cannot be resolved through discussion will be referred to a third reviewer. To enhance the transparency and credibility of the synthesis, an audit trail documenting coding decisions, theme development and analytical interpretations will be maintained throughout the review process (S2 Appendix). Where appropriate, similarities and differences in caregivers’ perspectives will be explored across different types of AI-enabled technologies and care settings to identify contextual factors that may influence the acceptance, concerns and expectations of family and informal caregivers.

### Assessment of confidence in the review findings

Confidence in each review finding will be assessed using the Grading of Recommendations Assessment, Development and Evaluation-Confidence in the Evidence from Reviews of Qualitative Research (GRADE-CERQual) approach [42]. GRADE-CERQual is a widely recognized framework for assessing the level of confidence that can be placed in findings generated from qualitative evidence syntheses and is recommended for use alongside qualitative systematic reviews [43].

The confidence in each review finding will be evaluated across four domains: (1) methodological limitations of the contributing primary studies, (2) coherence of the review finding, (3) adequacy of the data supporting the finding, and (4) relevance of the contributing studies to the review question [44]. This tool is presented in S3 Appendix. Judgments across these four domains will be used to determine an overall level of confidence for each review finding, which will be classified as high, moderate, low or very low confidence, in accordance with GRADE-CERQual guidance.

The CERQual assessments will be undertaken separately to evaluate confidence in the review findings rather than the quality of individual studies. The CERQual assessment will be conducted independently by two reviewers following completion of the thematic synthesis. Any disagreements will be resolved through discussion, and where necessary, consultation with a third reviewer. Moreover, a Summary of Qualitative Findings (SoQF) table will be prepared to present each review finding, the corresponding CERQual assessment and the rationale for the confidence judgement, thereby enhancing the transparency and interpretability of the review findings [45]. The Summary of Qualitative Findings (SoQF) table will include each analytical theme, the contributing studies, the CERQual assessment, and an explanation of the judgement to facilitate transparent interpretation of the review findings.

## Discussion

Artificial intelligence is expected to play an increasingly important role across the dementia care pathway, from early detection and clinical decision support to home-based monitoring and personalized care [46,47]. However, the successful implementation of AI-enabled dementia care depends not only on technological performance but also on whether it is accepted, trusted and meaningfully integrated into the everyday lives of people living with dementia and their caregivers. Family and informal caregivers are central to many decisions regarding the adoption, use and continued engagement with AI technologies, yet their perspectives remain fragmented across diverse qualitative studies [15]. By systematically synthesizing caregivers’ acceptance, concerns and expectations, this review aims to generate implementation-relevant evidence that extends beyond the evaluation of individual AI technologies and contributes to a more comprehensive understanding of the human factors influencing AI implementation in dementia care.

A key strength of this review is its focus on AI-enabled dementia care rather than on a single category of digital technology. Previous qualitative evidence syntheses have primarily examined caregivers’ experiences with specific interventions, such as socially assistive robots or supportive mobile applications [15,17]. While these reviews have generated valuable insights into particular technologies, they do not fully capture caregivers’ perspectives across the broader and rapidly evolving landscape of AI-enabled dementia care. By integrating evidence from diverse AI applications, this review will identify common themes related to caregivers’ acceptance, concerns and expectations, while also exploring contextual factors that may influence these perspectives across different technologies and care settings. Such an approach is expected to provide transferable insights that are relevant to the future implementation of AI in dementia care rather than to individual technologies alone.

The findings of this review may have implications for multiple stakeholders involved in the development and implementation of AI-enabled dementia care. For healthcare professionals, a better understanding of caregivers’ perspectives may support more effective communication and shared decision-making when introducing AI into dementia care. For technology developers, the synthesis may help identify user priorities, unmet needs and ethical concerns that should be considered during the design of caregiver-centred AI solutions. For policymakers and healthcare organizations, the review may provide evidence to inform implementation strategies, governance frameworks and policies that promote the safe, trustworthy and equitable integration of AI into dementia care. In addition, the review will identify areas where qualitative evidence remains limited, thereby informing priorities for future research.

Several limitations should be acknowledged. The review will include only peer-reviewed studies published in English, which may result in the omission of relevant evidence reported in other languages or unpublished sources. Furthermore, because AI technologies are evolving rapidly, new qualitative evidence may emerge after completion of the review. Nevertheless, the review will adopt a rigorous and transparent methodology, including a comprehensive search strategy, independent study selection, critical appraisal using the Joanna Briggs Institute methodology, thematic synthesis, and assessment of confidence in the review findings using GRADE-CERQual. By synthesizing qualitative evidence on family and informal caregivers’ acceptance, concerns and expectations, this review seeks to support the development, implementation and governance of AI-enabled dementia care that is not only technologically innovative but also trustworthy, ethically responsible and responsive to the needs and values of families caring for people living with dementia.

## Supporting information

S1 Checklist PRISMA-P (Preferred Reporting Items for Systematic review and Meta-Analysis Protocols) 2015 checklist: recommended items to address in a systematic review protocol*. (DOCX)

S1 Table JBI Critical Appraisal Checklist for Qualitative Research. (DOCX)

S2 Table Data extraction form for included qualitative studies. (DOCX)

S1 Appendix Search strategy for PubMed. (DOCX)

S2 Appendix Planned audit trail framework for qualitative data synthesis. (DOCX)

S3 Appendix Planned approach for assessing confidence in qualitative review findings using GRADE-CERQual. (DOCX)
