# Supplementary material for: Family and informal caregivers’ acceptance, concerns and expectations regarding artificial intelligence-enabled dementia care: a protocol for qualitative evidence synthesis
Source: PLoS One. 2026 Oct 7;21(10):e0360163. doi: 10.1371/journal.pone.0360163 (PMC13645028; doi:10.1371/journal.pone.0360163)
Supplement: Supplementary file 2 [file pone.0360163.s002.docx]

**S1 Table. JBI Critical Appraisal Checklist for Qualitative Research**

| **No.** | **Appraisal criterion** | **Response options** | **Study1** | **Study2** | **......** |
| --- | --- | --- | --- | --- | --- |
| 1 | Is there congruity between the stated philosophical perspective and the research methodology? | Yes / No / Unclear / Not applicable |  |  |  |
| 2 | Is there congruity between the research methodology and the research question or objectives? | Yes / No / Unclear / Not applicable |  |  |  |
| 3 | Is there congruity between the research methodology and the methods used to collect data? | Yes / No / Unclear / Not applicable |  |  |  |
| 4 | Is there congruity between the research methodology and the representation and analysis of data? | Yes / No / Unclear / Not applicable |  |  |  |
| 5 | Is there congruity between the research methodology and the interpretation of results? | Yes / No / Unclear / Not applicable |  |  |  |
| 6 | Is there a statement locating the researcher culturally or theoretically? | Yes / No / Unclear / Not applicable |  |  |  |
| 7 | Is the influence of the researcher on the research, and vice versa, addressed? | Yes / No / Unclear / Not applicable |  |  |  |
| 8 | Are participants, and their voices, adequately represented? | Yes / No / Unclear / Not applicable |  |  |  |
| 9 | Is the research ethical according to current criteria? | Yes / No / Unclear / Not applicable |  |  |  |
| 10 | Are the conclusions drawn in the research report supported by the analysis, or interpretation, of the data? | Yes / No / Unclear / Not applicable |  |  |  |
