# Supplementary material for: Family and informal caregivers’ acceptance, concerns and expectations regarding artificial intelligence-enabled dementia care: a protocol for qualitative evidence synthesis
Source: PLoS One. 2026 Oct 7;21(10):e0360163. doi: 10.1371/journal.pone.0360163 (PMC13645028; doi:10.1371/journal.pone.0360163)
Supplement: Supplementary file 3 [file pone.0360163.s003.docx]

**S2 table. Data extraction form for included qualitative studies**

| **Category** | **Data extraction items** |  |  |  |
| --- | --- | --- | --- | --- |
| **Study identification** |  | **study 1** | **study 2** | **...** |
| First author |  |  |  |  |
| Year of publication |  |  |  |  |
| Country/region where the study was conducted |  |  |  |  |
| Study setting | (e.g., community, home care, healthcare facility, dementia care organisation) |  |  |  |
| **Study aims and characteristics** |  |  |  |  |
| Study aim/objective |  |  |  |  |
| Research question(s) (if reported) |  |  |  |  |
| Study design | (e.g., qualitative descriptive study, phenomenology, grounded theory, ethnography) |  |  |  |
| Qualitative methodology/framework |  |  |  |  |
| Theoretical framework (if reported) |  |  |  |  |
| **Participants and caregiving characteristics** |  |  |  |  |
| Participant group | (family caregiver/informal caregiver/spouse/adult child/other relatives) |  |  |  |
| Number of participants |  |  |  |  |
| Participant characteristics | (age, sex, relationship with person living with dementia, caregiving duration, caregiving role, etc., if reported) |  |  |  |
| **AI-enabled dementia care characteristics** |  |  |  |  |
| Type of AI-enabled technology | (e.g., conversational AI, generative AI, machine learning-based systems, predictive analytics, intelligent monitoring systems, AI-enabled robots) |  |  |  |
| Description of the AI technology/intervention |  |  |  |  |
| Intended purpose of AI application | (e.g., monitoring, decision support, communication, companionship, symptom management, personalised care) |  |  |  |
| Care context of AI use | (home/community/clinical/other settings) |  |  |  |
| Level of caregiver involvement in AI use | (direct user, decision-maker, supporter, observer, other) |  |  |  |
| **Data collection and analysis** |  |  |  |  |
| Data collection method | (individual interviews/focus groups/observations/open-ended survey responses/other) |  |  |  |
| Data analysis method |  |  |  |  |
| **Qualitative findings related to caregivers’ perspectives** |  |  |  |  |
| Findings related to acceptance of AI-enabled dementia care | (e.g., willingness to use, perceived usefulness, trust, perceived compatibility with caregiving needs) |  |  |  |
| Findings related to concerns regarding AI-enabled dementia care | (e.g., privacy, safety, ethical issues, reduced human contact, technological barriers, responsibility concerns) |  |  |  |
| Findings related to expectations regarding AI-enabled dementia care | (e.g., desired functions, support needs, preferred design features, future expectations) |  |  |  |
| Other relevant caregiver perspectives | (e.g., perceived benefits, challenges, facilitators, barriers, emotional responses) |  |  |  |
| Authors’ interpretation of findings |  |  |  |  |
| **Reviewer interpretation and synthesis preparation** |  |  |  |  |
| Initial codes generated from findings |  |  |  |  |
| Potential descriptive themes |  |  |  |  |
| Potential analytical themes |  |  |  |  |
| Notes for thematic synthesis |  |  |  |  |
