# Supplementary material for: Family and informal caregivers’ acceptance, concerns and expectations regarding artificial intelligence-enabled dementia care: a protocol for qualitative evidence synthesis
Source: PLoS One. 2026 Oct 7;21(10):e0360163. doi: 10.1371/journal.pone.0360163 (PMC13645028; doi:10.1371/journal.pone.0360163)
Supplement: Supplementary file 4 [file pone.0360163.s004.docx]

**S1 Appendix. Search strategy for PubMed**

| 1 | #1 | ("Cognitive Impairment"[Mesh] OR "Dementia"[Mesh] OR "Alzheimer Disease"[Mesh] OR cognit* impair*[tiab] OR cognit* declin*[tiab] OR dement*[tiab] OR alzheimer*[tiab]) |
| --- | --- | --- |
| 2 | #2 | ("Caregivers"[Mesh] OR carer*[tiab]) OR (caregiv*[tiab) OR (care-giv*[tiab]) OR "informal caregiver*"[tiab] OR (famil*[tiab]) OR (relative*[tiab]) OR (relatives or spouse*[tiab] OR "adult child*"[tiab]) |
| 3 | #3 | ("Aged"[Mesh] OR older adult*[tiab] OR elderly[tiab] OR older people[tiab])  ("Artificial Intelligence"[Mesh] OR "artificial intelligence"[tiab] OR "machine learning"[Mesh] OR "machine learning"[tiab] OR "deep learning"[tiab] OR "generative AI"[tiab] OR "large language model*"[tiab] OR "conversational AI"[tiab] OR chatbot*[tiab] OR (intelligent[tiab] AND monitor*[tiab]) OR (  predict*[tiab] AND analytic*[tiab]) OR "socially assistive robot*"[tiab] OR "assistive robot*"[tiab]) |
| 4 | #4 | #1 AND #2 AND #3 |

Note: “*” to include all derivatives of that word or concept.
