# Supplementary material for: Family and informal caregivers’ acceptance, concerns and expectations regarding artificial intelligence-enabled dementia care: a protocol for qualitative evidence synthesis
Source: PLoS One. 2026 Oct 7;21(10):e0360163. doi: 10.1371/journal.pone.0360163 (PMC13645028; doi:10.1371/journal.pone.0360163)
Supplement: Supplementary file 5 [file pone.0360163.s005.docx]

**S2 Appendix. Planned audit trail framework for qualitative data synthesis**

| **Stage of synthesis** | **Audit trail items to be documented** | **Purpose** |
| --- | --- | --- |
| Data familiarization | Notes on repeated reading of extracted findings, initial observations and contextual considerations | To document researchers’ engagement with the qualitative evidence |
| Initial coding | Extracted findings, supporting participant quotations, initial codes generated, coding rationale | To maintain transparency between original evidence and generated codes |
| Development of descriptive themes | Related codes grouped together, rationale for grouping, preliminary theme descriptions | To document how patterns across studies are identified |
| Development of analytical themes | Interpretation beyond descriptive themes, theoretical/conceptual insights, relationship between themes | To demonstrate how higher-level interpretations are developed |
| Theme refinement | Decisions regarding merging, separating or excluding themes and reasons for changes | To document analytical decisions throughout synthesis |
| Reviewer discussions | Disagreements identified, discussions undertaken and decisions reached | To enhance credibility and consistency between reviewers |
| Final synthesis | Final themes, supporting studies, illustrative quotations and interpretation | To provide traceability from evidence to review findings |
