# Supplementary material for: Family and informal caregivers’ acceptance, concerns and expectations regarding artificial intelligence-enabled dementia care: a protocol for qualitative evidence synthesis
Source: PLoS One. 2026 Oct 7;21(10):e0360163. doi: 10.1371/journal.pone.0360163 (PMC13645028; doi:10.1371/journal.pone.0360163)
Supplement: Supplementary file 6 [file pone.0360163.s006.docx]

**S3 Appendix. Planned approach for assessing confidence in qualitative review findings using GRADE-CERQual**

| **CERQual component** | **Assessment considerations in this review** |
| --- | --- |
| Methodological limitations | Judged based on the results of the JBI Critical Appraisal Checklist for Qualitative Research. Limitations affecting credibility, data collection, analysis, reflexivity and ethical reporting will be considered. |
| Coherence | Assessed by examining how well the qualitative data support the review finding and whether findings are consistent across included studies. |
| Adequacy of data | Assessed according to the richness and quantity of data contributing to each review finding, including the number and depth of supporting quotations or observations. |
| Relevance | Assessed by considering the extent to which included studies address the review question, including similarities and differences in participants, settings and AI-enabled dementia care contexts. |
| Overall confidence judgement | Determined by integrating assessments across all four domains and classified as high, moderate, low or very low confidence. |
